# Evaluation of the value of conventional and unconventional lipid parameters for predicting the risk of diabetes in a non-diabetic population

**DOI:** 10.1186/s12967-022-03470-z

**Published:** 2022-06-11

**Authors:** Guotai Sheng, Maobin Kuang, Ruijuan Yang, Yanjia Zhong, Shuhua Zhang, Yang Zou

**Affiliations:** 1 Department of Cardiology, Jiangxi Provincial People’s Hospital, Nanchang, 330006 Jiangxi China; 2 Medical College of Nanchang University, Nanchang, 330006 Jiangxi China; 3 Department of Endocrinology, Jiangxi Provincial People’s Hospital, Nanchang, 330006 Jiangxi China; 4 Jiangxi Provincial People’s Hospital, Jiangxi Cardiovascular Research Institute, Nanchang, 330006 Jiangxi China

**Keywords:** Prediction, Unconventional lipid parameters, Lipid ratios, Diabetes, Conventional lipid parameters

## Abstract

**Background:**

Conventional and unconventional lipid parameters are associated with diabetes risk, the comparative studies on lipid parameters for predicting future diabetes risk, however, are still extremely limited, and the value of conventional and unconventional lipid parameters in predicting future diabetes has not been evaluated. This study was designed to determine the predictive value of conventional and unconventional lipid parameters for the future development of diabetes.

**Methods:**

The study was a longitudinal follow-up study of 15,464 participants with baseline normoglycemia. At baseline, conventional lipid parameters such as low-density lipoprotein cholesterol (LDL-C), triglyceride (TG), total cholesterol (TC), high-density lipoprotein cholesterol (HDL-C) were measured/calculated, and unconventional lipid parameters such as non-HDL-C, remnant cholesterol (RC), LDL/HDL-C ratio, TG/HDL-C ratio, non-HDL/HDL-C ratio, TC/HDL-C ratio and RC/HDL-C ratio were calculated. Hazard ratio (HR) and 95% confidence interval (CI) were estimated by Cox proportional hazard regression adjusting for demographic and diabetes-related risk factors. The predictive value and threshold fluctuation intervals of baseline conventional and unconventional lipid parameters for future diabetes were evaluated by the time-dependent receiver operator characteristics (ROC) curve.

**Results:**

The incidence rate of diabetes was 3.93 per 1000 person-years during an average follow-up period of 6.13 years. In the baseline non-diabetic population, only TG and HDL-C among the conventional lipid parameters were associated with future diabetes risk, while all the unconventional lipid parameters except non-HDL-C were significantly associated with future diabetes risk. In contrast, unconventional lipid parameters reflected diabetes risk better than conventional lipid parameters, and RC/HDL-C ratio was the best lipid parameter to reflect the risk of diabetes (HR: 6.75, 95% CI 2.40–18.98). Sensitivity analysis further verified the robustness of this result. Also, time-dependent ROC curve analysis showed that RC, non-HDL/HDL-C ratio, and TC/HDL-C ratio were the best lipid parameters for predicting the risk of medium-and long-term diabetes.

**Conclusions:**

Unconventional lipid parameters generally outperform conventional lipid parameters in assessing and predicting future diabetes risk. It is suggested that unconventional lipid parameters should also be routinely evaluated in clinical practice.

**Supplementary Information:**

The online version contains supplementary material available at 10.1186/s12967-022-03470-z.

## Background

The high prevalence of diabetes and its extensive complications have created a huge disease burden for mankind, which is one of the leading causes of death and disability worldwide [1–3]. Diabetes is known to be associated with a series of interrelated abnormalities of plasma lipids and lipoproteins. Diabetic individuals generally exhibit a pattern of atherogenic risk factors compared with individuals without diabetes [4]. Among them, metabolic disturbances of triglyceride-rich lipoproteins are thought to be key to the pathophysiology of this lipids-induced atherosclerosis, including increased hepatic secretion of VLDL and impaired clearance of VLDL and gut-synthesized chylomicrons [4–7]. Clinically, the main manifestations in conventional lipids are decreased HDL-C levels and increased TG levels [4, 8], while that in unconventional lipids are elevated levels of RC, TC/HDL ratio, non-HDL-C, TG/HDL-C ratio, non-HDL/HDL-C ratio, and LDL/HDL-C ratio [9–12]. These dyslipidemia characteristics are associated with an increased risk of cardiovascular disease [13–15], which is the leading cause of death in patients with type 2 diabetes [16]. Therefore, early identification of the above-mentioned diabetic dyslipidemia manifestations can effectively prevent and intervene the risk of diabetes, which is all-important for reducing the incidence of diabetes and diabetes-related mortality. In recent years, a series of studies have been carried out on the relationship between conventional and unconventional lipid parameters and the risk of diabetes [8–12, 17, 18]; on the whole, unconventional lipid parameters appear to have good application value, and they reveal excellent performance in the assessment and prediction of diabetes risk. However, comparative studies on lipid parameters for predicting future diabetes risk are still relatively limited [10, 18, 19], and the value of conventional and unconventional lipid parameters in predicting future diabetes has not been evaluated. To address this question, in the current study, we investigated the independent association and predictive value of conventional and unconventional lipid parameters (addition the RC/HDL-C ratio on the basis of previous studies) with the future risk of diabetes based on NAGALA large longitudinal cohort study data.

## Methods

### Data sources and research population

**Fig. 1:**
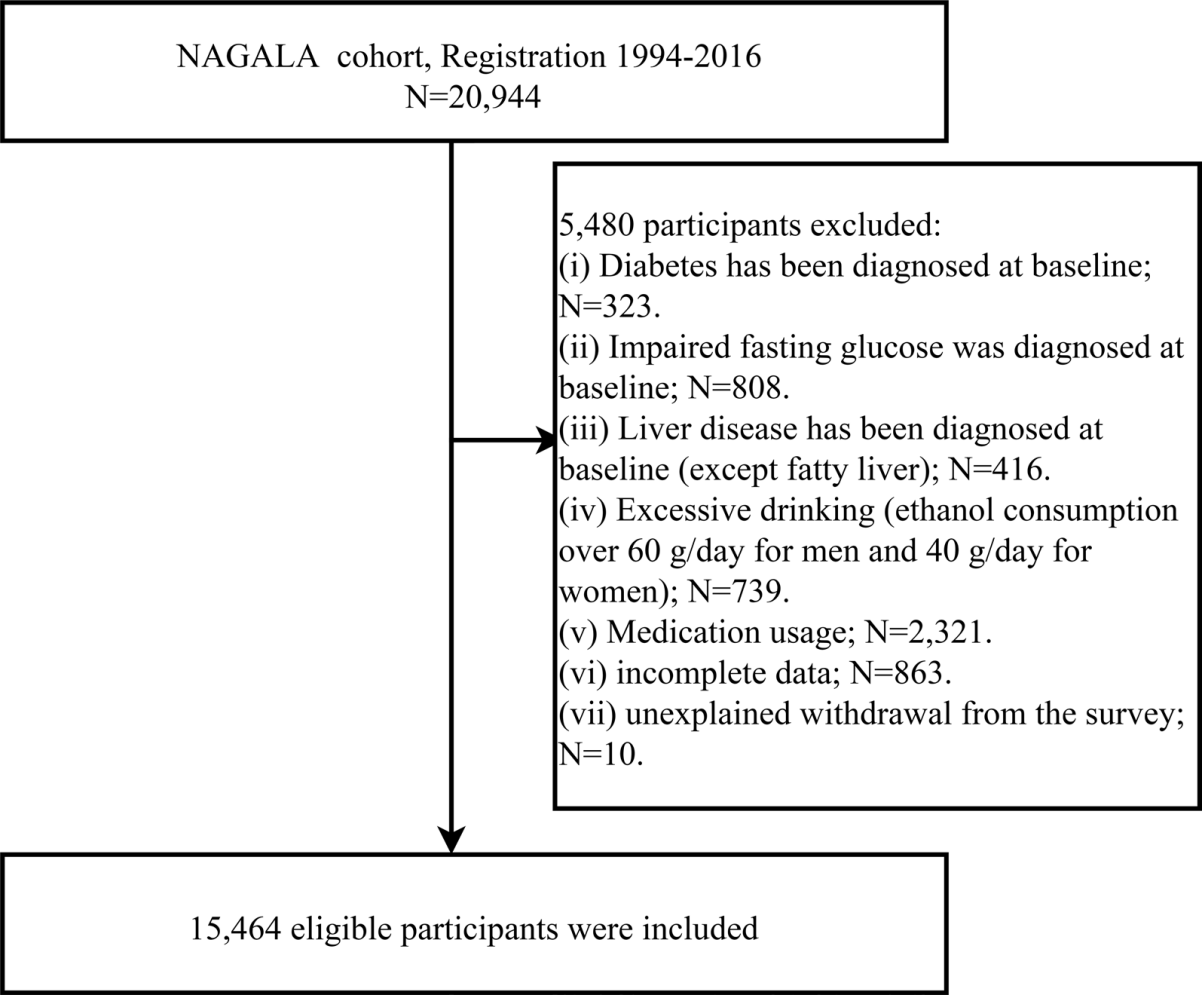
Study profile

In this current longitudinal analysis, we used NAGALA cohort data from 1994 to 2016 to investigate the predictive value of conventional and unconventional lipid parameters for future diabetes risk. The NAGALA cohort data has been uploaded, by Professor Okamura, to the Dryad database for public sharing [20], and a detailed description of the research design has been published previously [21]. In short, NAGALA is a longitudinal cohort study based on the health check-up population, which began in 1994 and continuously recruited people who took health examinations at Murakami Memorial Hospital (follow-up response rate was approximately 60%) to assess non-communicable diseases and their related risk factors. In the current research dataset, 12,498 male and 8446 female participants who registered to participate in the research from 1994 to 2016 were included, and the last visit was on December 31, 2016. According to the purpose of the study, we excluded participants with diabetes, impaired fasting glucose, liver disease, excessive drinking [22], incomplete data, baseline medication, and unexplained withdrawal from the survey. Figure 1 shows the current research population screening process.

### Ethical approval and consent to participation

In the initial survey, the Murakami Memorial Hospital Institutional Ethics Review Board approved the NAGALA cohort study, and informed written consent was obtained from each participant for the use of their data (IRB2018-09-01) [21]. This study was a secondary analysis of data from the NAGALA cohort. The research scheme has been approved by the Ethics Review Committee of Jiangxi Provincial People’s Hospital (IRB2021-066), and the entire process followed the Helsinki Declaration. See STROBE statement in Additional file 1: Text S1.

### Population and lifestyle data

Information on age, sex, smoking and drinking, medication history, disease history, and exercise habits was self-reported. Blood pressure, weight, waist circumference, height, and body mass index (BMI) were measured according to the standard protocols. In terms of exercise habits, participants were assessed for the frequency of physical exercise per week, and more than one time per week was considered an exercise habit. Drinking status was assessed based on the amount and type of weekly alcohol consumption by participants in the past month and participants were classified as non/small drinkers, light drinkers, moderate drinkers, and heavy drinkers [22]. For smoking status, participants were classified as nonsmokers, past smokers, and current smokers.

### Determination and calculation of biomarkers

Venous blood samples were drawn from study participants after fasting for > 8 h, and the concentrations of HDL-C, gamma-glutamyl transferase (GGT), fasting plasma glucose (FPG), TC, aspartate aminotransferase (AST), TG, hemoglobin A1c (HbA1c), and alanine aminotransferase (ALT) were determined by the automatic biochemical analyzer in a standard laboratory.

The lipid parameters were calculated as follows:

TG/HDL-C ratio = TG/HDL-C [10];

TC/HDL-C ratio = TC/HDL-C [10];

Non-HDL-C = TC − HDL-C [11];

LDL-C = 90% non-HDL-C − 10%TG [23];

LDL/HDL-C ratio = LDL-C/HDL-C [12];

Non-HDL/HDL-C ratio = non-HDL-C/HDL-C [23];

RC = Non-HDL-C − LDL-C [24];

RC/HDL-C ratio = RC/HDL-C [25].

### Determination of diabetes fatty liver, and metabolic score for insulin resistance (METS-IR)

The diagnosis of diabetes was based on follow-up physical examination information of the participants. Referring to the criteria of the American Diabetes Association [26], participants were diagnosed with diabetes if they measured HbA1c ≥ 6.5% or FPG ≥ 7.0 mmol/L during follow-up. Additionally, the diabetes diagnosis self-reported by the participants during follow-up was also included in the analysis.

Fatty liver was diagnosed by gastroenterologists based on the following features of abdominal Doppler ultrasound: vascular blurring, liver brightness, deep attenuation, and hepatorenal echo contrast [27].

METS-IR: Ln [BMI × (TG + 2FPG)]/[Ln(HDL-C)] [28].

### Statistical analysis

Descriptive data were expressed as the count (percentage) of categorical variables and the median (interquartile range) or mean (standard deviation) of continuous variables. Pearson chi-square test for categorical variables and the Mann–Whitney U test or Student’s t-test for continuous variables were used to compare the differences between groups. In addition, to further quantify the differences between groups, we also calculated the standardized differences between groups (the difference > 10% was considered significant) [29, 30].

Several ancillary analyses were carried out before evaluating the association of lipid parameters with diabetes risk. Firstly, the Pearson correlation coefficient was calculated to evaluate the correlation of lipid parameters with METS-IR, in which the non-normal continuous variables were analyzed after the Box-Cox normal transformation [31]. Secondly, the collinearity of covariates was checked by multiple linear regression, in which covariates with a variance inflation factor (VIF)  > 5 would not be included in the subsequent multivariate adjustment model [32]. Thirdly, the proportional hazard assumption was checked by visual inspection of the Kaplan–Meier (KM) curve.

The associations and magnitudes of associations between baseline conventional and unconventional lipid parameter levels and follow-up endpoints were estimated using Cox proportional hazard regression model, and the corresponding HR and 95% CI were recorded. To systematically consider the influence of confounding factors on the outcome, we evaluated four multivariate Cox regression models based on epidemiology [33]. In the first model (Model 1), the effects of the principal basic demographic data (age, sex, and BMI) on the main endpoints were considered. On this basis, the second model further incorporated important risk factors such as exercise habits, smoking status, drinking status, and fatty liver (Model 2). Model 3 further adjusted the related parameters of blood pressure and glucose based on model 2. To fully consider the potential role of covariates, we took the fourth model as the final model to adjust all non-collinear variables except lipid parameters (Model 4).

We conducted three sensitivity analyses based on model 4 to assess the robustness of the relationship between lipid parameters and diabetes risk. Sensitivity-1: exclude people diagnosed with diabetes within 2 years of entry into the study cohort to reduce the potential impact of reverse causality. Sensitivity-2: to minimize confounding in the relationship between lipid parameters and diabetes due to baseline fatty liver, we limited the analysis to people without fatty liver. Sensitivity-3: participants with high blood pressure at baseline were excluded because hypertension was associated with an increased risk of diabetes.

Based on the results of the association analysis, we further drew time-dependent ROC curves and calculated the area under the 3-year, 6-year, 9-year, and 12-year time-dependent ROC curves to quantify the accuracy of lipid parameters for predicting the future diabetes risk, and calculated the corresponding best threshold. Additionally, to verify the validity of best thresholds of lipid parameters for predicting future diabetes risk, we also fitted the shape of the dose–response correlation between lipid parameters and diabetes risk using a 4-knots restricted cubic splines (RCS, based on model 4) analysis nested in Cox regression [34]. By visually assessing the shape of the curve, the value of the lipid parameter corresponding to HR = 1 was determined as the best threshold. All analyses were done using the statistical programming language R (version 3.4.3; www.r-project.org) and Empower (R) (version 3.4.3; X&Y Solutions, Inc., Boston, MA, USA). All tests were two-tailed and the significance was set to *P* < 0.05.

## Results

### Baseline characteristics

The study population consisted of 15,464 baseline diabetes-free participants, with a mean age of 43.7 (8.9) years. During a mean follow-up of 6.13 years (min.–max.: 0.46–13.14 years), 373 participants were diagnosed with new-onset diabetes (3.93 per 1000 person-years). Differences in baseline characteristics of the study population were reported in Table 1 according to whether or not diabetes will develop in the future. As expected, there were already significant differences at baseline among people with or without diabetes in the future (All *P* < 0.05). Notably, those without diabetes showed the greatest differences in baseline glucose metabolism (FPG, HbA1c, METS-IR) compared with those who developed diabetes during follow-up (Standardized difference > 100%). Secondly, judging from the demographic characteristics of the data, there were big differences in weight, waist circumference, and BMI between the two groups (Standardized difference: 76–92%). Among the lipid parameters, except non-HDL-C, LDL-C, and TC, all conventional and unconventional lipid parameters have shown great differences in the early stage of physical examination (Standardized difference > 70%). Finally, it is also worth mentioning that the difference between the two groups in the population with fatty liver at baseline should also be noted (Standardized difference: 99%). These findings suggested that early physical examination and assessment of these widely differing baseline characteristics may be useful for predicting future diabetes risk.

**Table 1:** Baseline demographic, lifestyle, and laboratory characteristics in participants with and without diabetes

	Non-diabetic	Diabetes	Standardized difference, % (95% CI)	*P *value
Participants, n	15,091	373		
Sex			49 (39, 59)	< 0.001
Women	6947 (46.03%)	87 (23.32%)		
Men	8144 (53.97%)	286 (76.68%)		
Age, years	42.00 (37.00–50.00)	46.00 (41.00–53.00)	40 (30, 51)	< 0.001
Height, m	1.65 (0.08)	1.67 (0.09)	19 (9, 29)	< 0.001
Weight, kg	60.41 (11.48)	69.84 (13.32)	76 (65, 86)	< 0.001
BMI, kg/m^2^	22.04 (3.07)	25.03 (3.82)	86 (76, 97)	< 0.001
WC, cm	76.26 (8.97)	85.08 (10.20)	92 (82, 102)	< 0.001
ALT, U/L	17.00 (13.00–23.00)	24.00 (18.00–39.00)	67 (56, 77)	< 0.001
AST, U/L	17.00 (14.00–21.00)	20.00 (16.00–26.00)	44 (34, 55)	< 0.001
GGT, U/L	15.00 (11.00–22.00)	24.00 (17.00–36.00)	47 (37, 58)	< 0.001
HDL-C, mmol/L	1.42 (1.17–1.71)	1.13 (0.96–1.32)	77 (66, 87)	< 0.001
TC, mmol/L	5.12 (0.86)	5.43 (0.90)	35 (25, 46)	< 0.001
TG, mmol/L	0.72 (0.49–1.11)	1.21 (0.86–1.93)	73 (62, 83)	< 0.001
LDL-C, mmol/L	3.03 (2.55–3.55)	3.46 (2.93–3.95)	52 (42, 63)	< 0.001
Non-HDL-C, mmol/L	3.59 (3.00–4.23)	4.20 (3.57–4.82)	65 (54, 75)	< 0.001
RC, mmol/L	0.53 (0.43–0.67)	0.71 (0.58–0.91)	80 (70, 91)	< 0.001
TC/HDL-C ratio	3.50 (2.86–4.39)	4.71 (3.86–5.78)	87 (77, 97)	< 0.001
TG/HDL-C ratio	0.50 (0.30–0.89)	1.09 (0.64–1.93)	74 (63, 84)	< 0.001
LDL/HDL-C ratio	2.12 (1.59–2.83)	3.01 (2.38–3.85)	83 (73, 93)	< 0.001
Non-HDL/HDL-C ratio	2.50 (1.86–3.39)	3.71 (2.86–4.78)	87 (77, 97)	< 0.001
RC/HDL-C ratio	0.53 (0.43–0.67)	0.71 (0.58–0.91)	78 (67, 88)	< 0.001
FPG, mmol/L	5.15 (0.41)	5.61 (0.36)	121 (111, 132)	< 0.001
HbA1c, %	5.16 (0.32)	5.53 (0.37)	107 (97, 118)	< 0.001
METS-IR	30.98 (6.36)	38.58 (7.74)	107 (97, 118)	< 0.001
SBP, mmHg	114.31 (14.91)	122.03 (15.59)	51 (40, 61)	< 0.001
DBP, mmHg	71.44 (10.47)	77.18 (10.23)	55 (45, 66)	< 0.001
Fatty liver	2518 (16.69%)	223 (59.79%)	99 (89, 109)	< 0.001
Exercise habits	2658 (17.61%)	51 (13.67%)	11 (1, 21)	0.048
Drinking status			21 (11, 31)	< 0.001
Non/small	11,539 (76.46%)	266 (71.31%)		
Light	1718 (11.38%)	40 (10.72%)		
Moderate	1323 (8.77%)	37 (9.92%)		
Heavy	511 (3.39%)	30 (8.04%)		
Smoking status			45 (35, 55)	< 0.001
None	8886 (58.88%)	145 (38.87%)		
Past	2875 (19.05%)	77 (20.64%)		
Current	3330 (22.07%)	151 (40.48%)		

Values were expressed as mean (SD) or medians (quartile interval) or n (%)

*BMI* body mass index; *WC* waist circumference; *ALT* alanine aminotransferase; *AST* aspartate aminotransferase; *GGT* gamma-glutamyl transferase; *HDL-C* high-density lipoprotein cholesterol; *TC* total cholesterol; *Non-HDL-C* non-high-density lipoprotein-cholesterol; *LDL-C* low density lipoprotein cholesterol; *TG* triglyceride; *RC* remnant cholesterol; *HbA1c* hemoglobin A1c; *FPG* fasting plasma glucose; *SBP* systolic blood pressure; *DBP* diastolic blood pressure; *METS-IR* metabolic score for insulin resistance

### Correlation between baseline conventional and unconventional lipid parameters and METS-IR

Pearson correlation analysis showed that all conventional and unconventional lipid parameters were correlated with METS-IR at baseline (Additional file 3: Table S1). In contrast, the lipid parameter/HDL-C ratio and HDL-C showed stronger statistical linear correlations with METS-IR than other lipid parameters (Pearson r = − 0.7139 for HDL-C; Pearson r = 0.7324 for TC/HDL-C ratio; Pearson r = 0.7447 for TG/HDL-C ratio; Pearson r = 0.7049 for LDL/HDL-C ratio; Pearson r = 0.7318 for non-HDL/HDL-C ratio; Pearson r = 0.7139 for RC/HDL-C ratio).

### Relationship between baseline conventional and unconventional lipid parameters and future diabetes

Before performing multivariate Cox regression analysis, our data passed the log-linear assumption and the proportional hazard assumption tests. Additional file 3: Table S2 shows the collinearity screening procedure according to VIF, and the collinear variables waist circumference, SBP, and weight will not be included in the following multivariable model. Additional file 2: Fig. S1 takes the ratio of RC/HDL-C as an example to show the KM curve corresponding to the quartiles of the RC/HDL-C ratio, and the results showed that there was no intersection between the KM curves.

Based on epidemiology, we ran four multivariate Cox regression models to evaluate the relationship of baseline conventional and unconventional lipid parameters with future diabetes risk (Table 2). In the models adjusted for demographic and lifestyle factors (Model 1 and Model 2), all lipid parameters were associated with future diabetes risk. However, the associations between LDL-C, non-HDL-C, and TC with the risk of diabetes disappeared after further consideration of the effects of blood pressure and glucose (Model 3). In the final model (Model 4), we adjusted all non-collinear variables except lipid parameters and obtained similar results to Model 3. Among the conventional lipid parameters, only TG and HDL-C were related to the risk of diabetes, while all unconventional lipid parameters except non-HDL-C were associated with the risk of diabetes. It is worth mentioning that by comparing the HR of lipid parameters corresponding to the risk of diabetes, we found that RC/HDL-C ratio can better reflect the risk of developing diabetes in the future than other lipid parameters (HR: 6.75, 95% CI 2.40–18.98).

**Table 2:** Association of baseline conventional and unconventional lipid parameters and future diabetes

	HR (95% CI)			
	Model 1	Model 2	Model 3	Model 4
HDL-C	0.32 (0.22, 0.47)	0.49 (0.33, 0.73)	0.55 (0.38, 0.81)	0.53 (0.36, 0.78)
TC	1.18 (1.05, 1.33)	1.13 (1.00, 1.28)	0.94 (0.83, 1.06)	0.92 (0.81, 1.04)
TG	1.48 (1.34, 1.63)	1.34 (1.20, 1.50)	1.21 (1.07, 1.36)	1.20 (1.06, 1.35)
LDL-C	1.27 (1.10, 1.46)	1.17 (1.01, 1.35)	0.94 (0.81, 1.09)	0.93 (0.80, 1.08)
Non-HDL-C	1.33 (1.19, 1.50)	1.21 (1.07, 1.37)	1.00 (0.88, 1.13)	0.99 (0.87, 1.12)
RC	4.55 (3.16, 6.55)	3.06 (2.03, 4.60)	1.72 (1.11, 2.67)	1.66 (1.07, 2.58)
TC/HDL-C ratio	1.34 (1.24, 1.44)	1.21 (1.12, 1.32)	1.11 (1.02, 1.21)	1.11 (1.02, 1.21)
TG/HDL-C ratio	1.34 (1.25, 1.42)	1.26 (1.16, 1.36)	1.19 (1.09, 1.30)	1.18 (1.08, 1.29)
Non-HDL/HDL-C ratio	1.34 (1.24, 1.44)	1.21 (1.12, 1.32)	1.11 (1.02, 1.21)	1.11 (1.02, 1.21)
LDL/HDL-C ratio	1.39 (1.26, 1.52)	1.24 (1.11, 1.37)	1.11 (1.00, 1.24)	1.12 (1.00, 1.24)
RC/HDL-C ratio	25.49 (9.69, 67.06)	8.13 (2.84, 23.24)	6.08 (2.17, 17.08)	6.75 (2.40, 18.98)

Model 1 adjusted for sex, age and BMI

Model 2 adjusted for sex, age, BMI, exercise habits, fatty liver, drinking status and smoking status

Model 3 adjusted for sex, age, BMI, exercise habits, fatty liver, drinking status, smoking status, SBP, FPG and HbA1c

Model 4 adjusted for sex, age, BMI, exercise habits, fatty liver, drinking status, smoking status, SBP, FPG, HbA1c, height, ALT, AST and GGT

*HR* hazard ratios; *CI* confidence interval; other abbreviations as in Table 1

### Sensitivity analysis

After adjusting for covariates based on model 4, the results of the three sensitivity analyses (Table 3) were consistent with the main results of Table 2: there were no significant correlations of LDL-C, non-HDL-C, TC with diabetes risk, while other lipid parameters were all significantly correlated with diabetes risk, and the HR of diabetes risk associated with RC/HDL-C ratio was higher than other lipid parameters.

**Table 3:** Adjusted hazard ratios and 95% confidence intervals for diabetes risk associated with the lipid parameters in different test populations: sensitivity analysis

	HR (95% CI)		
	Sensitivity-1	Sensitivity-2	Sensitivity-3
HDL-C	0.63 (0.41, 0.99)	0.49 (0.29, 0.82)	0.50 (0.34, 0.75)
TC	0.99 (0.86, 1.14)	0.89 (0.73, 1.08)	0.97 (0.85, 1.10)
TG	1.17 (1.01, 1.34)	1.24 (1.00, 1.52)	1.30 (1.15, 1.46)
LDL-C	1.00 (0.85, 1.19)	0.94 (0.75, 1.18)	0.98 (0.84, 1.14)
Non-HDL-C	1.04 (0.90, 1.20)	0.99 (0.81, 1.20)	1.04 (0.92, 1.19)
RC	1.64 (0.98, 2.74)	1.74 (0.82, 3.72)	2.25 (1.44, 3.53)
TC/HDL-C ratio	1.10 (1.00, 1.21)	1.17 (1.01, 1.35)	1.15 (1.05, 1.26)
TG/HDL-C ratio	1.14 (1.02, 1.27)	1.19 (1.03, 1.37)	1.23 (1.13, 1.34)
Non-HDL/HDL-C ratio	1.10 (1.00, 1.21)	1.17 (1.01, 1.35)	1.15 (1.05, 1.26)
LDL/HDL-C ratio	1.11 (0.98, 1.26)	1.20 (1.00, 1.44)	1.16 (1.03, 1.29)
RC/HDL-C ratio	3.69 (1.09, 12.47)	14.01 (2.75, 71.47)	7.73 (2.68, 22.28)

Note 1: (1) sensitivity-1: subjects with a follow-up of less than 3 years were excluded (n = 11,237); (2) sensitivity-2: subjects diagnosed with fatty liver at baseline were excluded. (n = 12,823); (3) sensitivity-3: excluding subjects whose SBP ≥ 140 mmHg or DBP ≥ 90 mmHg (n = 14,878);

Note 2: sensitivity-1 adjusted for sex, age, BMI, habits of exercise, drinking status, smoking status, fatty liver, SBP, FPG, HbA1c, height, ALT, AST and GGT. Sensitivity-2 adjusted for sex, age, BMI, habits of exercise, drinking status, smoking status, SBP, FPG, HbA1c, height, ALT, AST and GGT. Sensitivity-3 adjusted for sex, age, BMI, habits of exercise, drinking status, smoking status, fatty liver, FPG, HbA1c, height, ALT, AST and GGT

*HR* hazard ratios; *CI* confidence interval; other abbreviations as in Table 1

### Predictive value of conventional and unconventional lipid parameters for future diabetes risk

**Fig. 2:**
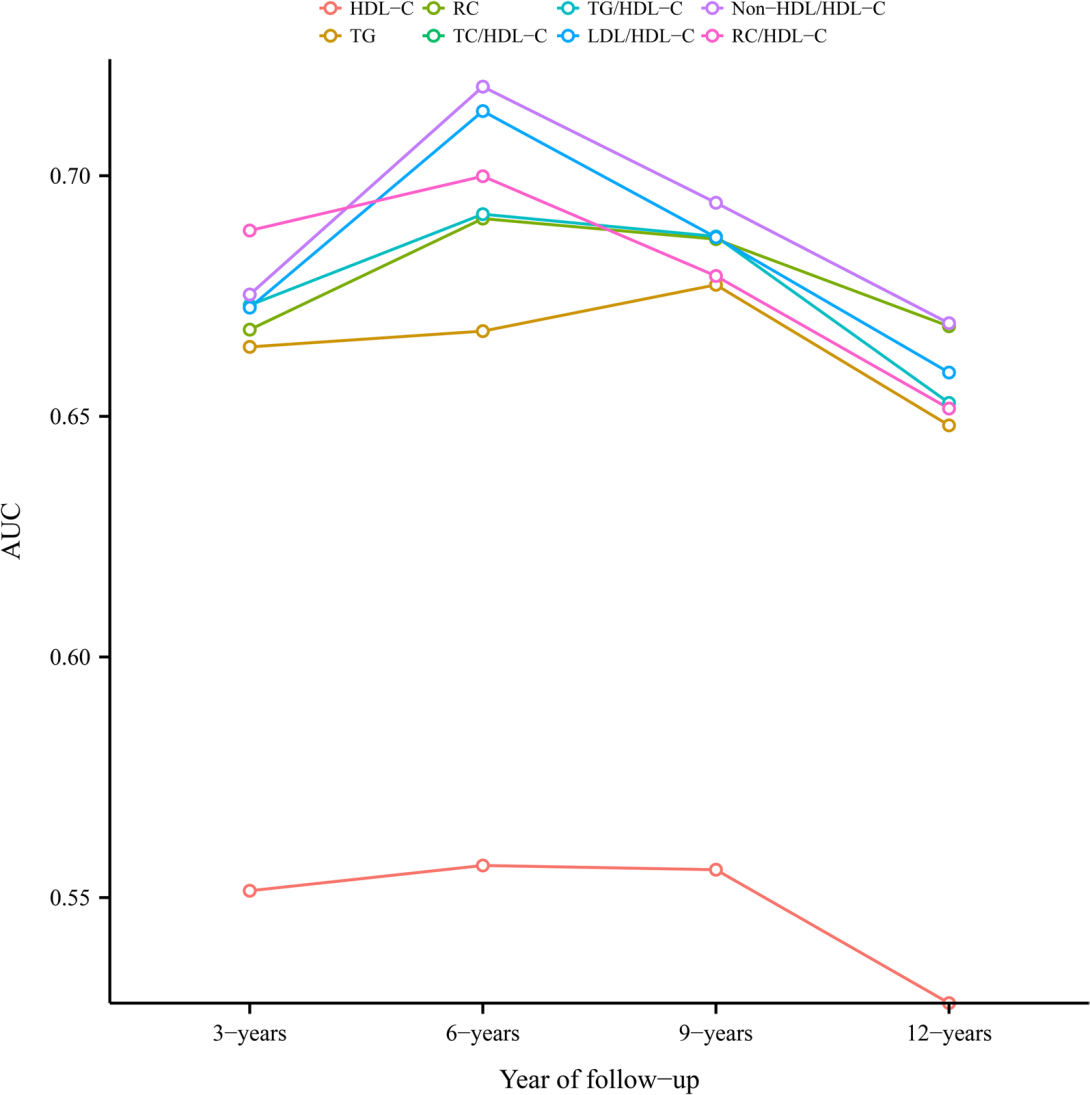
The area under the receiver operator characteristics curve of lipid parameters varying with time to predict the future risk of diabetes. *AUC* area under the curve

Time-dependent ROC curves were drawn to evaluate the predictive value of HDL-C, TG, and unconventional lipid parameters for future diabetes risk (Table 4). Figure 2 shows the area under the curve of these lipid parameters over time. Overall, the predictive value of all lipid parameters for diabetes risk decreased over time. Furthermore, relatively speaking, the predictive value of unconventional lipid parameters for the risk of diabetes was slightly higher than that of conventional lipid parameters. Among them, RC/HDL-C ratio had the best predictive value for predicting short-term diabetes risk, non-HDL/HDL-C ratio and LDL/HDL-C ratio had the best predictive value for the medium-term diabetes risk, while non-HDL/HDL-C ratio RC and TC/HDL-C ratio had the best predictive value for the medium-and long-term diabetes risk.

**Table 4:** Best threshold and areas under the time-dependent receiver operating characteristic curves for each lipid parameters predicting future diabetes risk

	3-years	6-years	9-years	12-years
	AUC (best threshold)	AUC (best threshold)	AUC (best threshold)	AUC (best threshold)
HDL-C	0.55142 (1.5025)	0.55667 (1.5025)	0.55579 (1.3602)	0.52807 (1.4792)
TG	0.66443 (0.8242)	0.66771 (0.8468)	0.67733 (0.8242)	0.64812 (0.8580)
RC	0.66802 (0.6280)	0.69109 (0.5832)	0.68682 (0.5705)	0.66872 (0.5578)
TC/HDL-C ratio	0.6753 (3.9741)	0.7185 (3.7134)	0.69438 (3.6975)	0.66934 (3.7351)
TG/HDL-C ratio	0.6731 (0.6567)	0.69201 (0.5983)	0.68738 (0.6118)	0.65279 (0.7276)
LDL/HDL-C ratio	0.67259 (2.3345)	0.71343 (2.3191)	0.6872 (2.2192)	0.6591 (2.3001)
Non-HDL/HDL-C ratio	0.67532 (2.9741)	0.71851 (2.7134)	0.69438 (2.6975)	0.66935 (2.7351)
RC/HDL-C ratio	0.68861 (0.3610)	0.69989 (0.3509)	0.67917 (0.3645)	0.65162 (0.3681)

*AUC* area under the curve; other abbreviations as in Table 1

### Threshold analysis of conventional and unconventional lipid parameters for predicting the risk of diabetes in the future

**Fig. 3:**
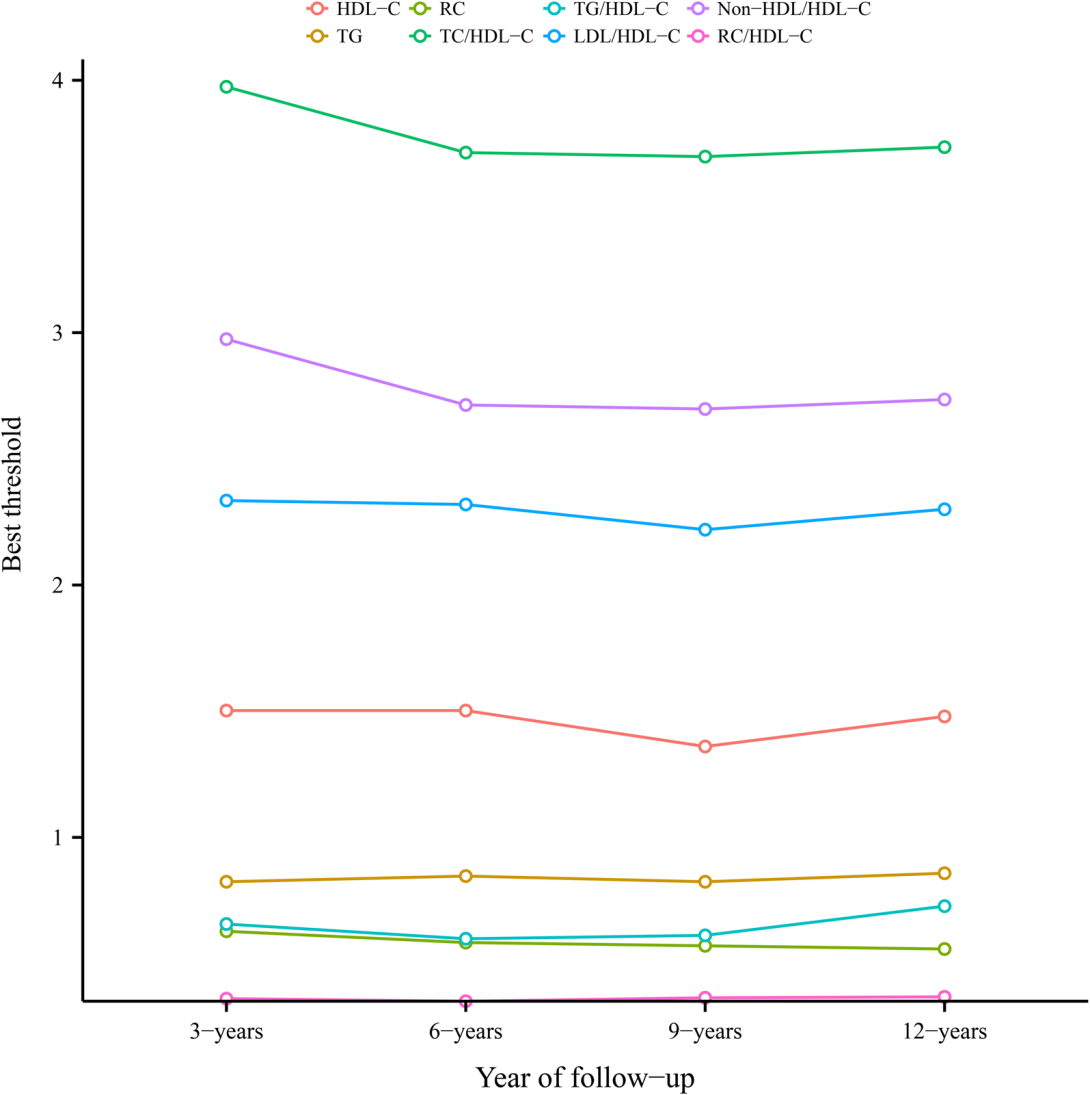
Threshold fluctuation of lipid parameters used to predict future diabetes risk

Using the time-dependent ROC curve, we also calculated the best thresholds of conventional and unconventional lipid parameters for predicting the risk of diabetes at 3, 6, 9, and 12 years. As can be seen from Fig. 3, despite the passage of time, the thresholds of all lipid parameters used to predict future diabetes risk only fluctuated within a narrow range (HDL-C: 1.3602–1.5025; TG: 0.8242–0.8580; RC: 0.5578–0.6280; TC/HDL-C ratio: 3.6975–3.9741; TG/HDL-C ratio: 0.5983–0.7276; LDL-HDL-C ratio: 2.2192–2.3191; Non-HDL/HDL-C ratio: 2.6975–2.9741; RC/HDL-C ratio: 0.3509–0.3681). Moreover, through RCS, we confirmed that the thresholds of HDL-C, TG, RC, TC/HDL-C ratio, TG/HDL-C ratio, LDL/HDL-C ratio, non-HDL/HDL-C ratio, and RC/HDL-C ratio were 1.46 mmol/L, 0.90 mmol/L, 0.58 mmol/L, 3.75, 0.71, 2.30, 2.75 and 0.36, respectively (Additional file 2: Fig. S2A–H). In the threshold analysis of RCS and ROC, the thresholds of all lipid parameters were within the stable fluctuation range, except for the slight differences in the threshold levels of TG.

## Discussion

There were four main findings in this longitudinal cohort study: (1) In the baseline non-diabetic population, only TG and HDL-C among the conventional lipid parameters were associated with future diabetes risk, while all the unconventional lipid parameters except non-HDL-C were significantly associated with the future risk of diabetes. (2) Compared with other conventional and unconventional lipid parameters, the RC/HDL-C ratio can better reflect the future diabetes risk. (3) Compared with conventional lipid parameters, unconventional lipid parameters were of higher value for predicting the diabetes risk in the future. (4) RC/HDL-C ratio had the best predictive value for the short-term diabetes risk, non-HDL/HDL-C ratio and LDL/HDL-C ratio for the short-to medium-term diabetes risk, while RC, non-HDL/HDL-C ratio, and TC/HDL-C ratio for the mid-to long-term and long-term diabetes risk.

Diabetes mellitus, one of the most rapidly growing chronic non-communicable diseases in this century, is a complex metabolic disease that presents with different subphenotypes according to various etiologies [35, 36]. The most common one is the metabolic syndrome-related phenotype, which is mainly characterized by dyslipidemia and obesity. Effective management of these complex lipid metabolic disorders and obesity is an important part of comprehensive diabetes prevention, treatment, and care, and can reduce the risk of cardiovascular morbidity and mortality [37]. The relationship between conventional lipid parameters HDL-C, TC, LDL-C, and TG and diabetes has been widely studied in the past, in which high concentrations of TG and low concentrations of HDL-C significantly increased diabetes risk has become the consensus of almost every scholar in the field of metabolism [37–39], but there is still some debate about the direct relationship between LDL-C, TC, and diabetes [10, 40, 41]. However, it should be mentioned that in terms of treatment, reducing LDL-C levels is still the primary task of lipid management in patients with diabetes [37], and multifaceted treatment strategies for hyperlipidemia, hyperglycemia, and hypertension can further improve the health status of diabetic patients [42]. In the current study, we also observed, from the comparison of baseline information, that compared with those who did not develop diabetes during the follow-up period, people with new-onset diabetes showed significant differences in all conventional and unconventional lipid parameters except non-HDL-C, LDL-C, and TC at baseline. Moreover, in regression analysis, when only adjusting for lifestyle and demographic data, all conventional lipid parameters were associated with future diabetes risk. In further covariation-adjusted models, the associations of LDL-C and TC with diabetes risk disappeared. A similar situation was also described in the work of Khaloo et al. [10]. This phenomenon suggested that LDL-C and TC may play a certain role in the risk of diabetes in the future, but this role may be weak and unstable compared with other lipid parameters.

Unconventional lipid parameters such as lipid ratio, RC, and non-HDL-C are hot topics in recent years. These lipid parameters are calculated by conventional lipid parameters according to certain formulas or direct division [10–12, 23–25]. At present, a series of epidemiological studies have been carried out using unconventional lipid parameters as novel markers in the endocrine system, cardio-cerebrovascular system, digestive system, respiratory system, and urinary system diseases [8–12, 43–48]. In general, unconventional lipid parameters improved the ability of conventional lipid parameters to assess and identify the risk of most diseases.

The relationship between conventional and unconventional lipid parameters and diabetes has been reported in many studies [8–12, 17, 18], but comparative studies on lipid parameters for predicting future diabetes are extremely limited. There were still some differences in the results of several longitudinal studies that have been published. Hadaegh et al. conducted the first comparative study in Iran in 2010 to evaluate the utility of conventional and unconventional lipid parameters to predict future diabetes risk [19]. They measured/calculated TG/HDL-C ratios, non-HDL-C, HDL-C, TC, and TG in 5,201 baseline diabetes-free subjects. After a median follow-up of 5.6 years, they found that the TG/HDL-C ratio was a better indicator of future diabetes risk in women, while the TC/HDL-C ratio was a better indicator of future diabetes risk in men. Subsequently, in 2011, South Korea’s Seo et al. conducted another longitudinal study of 5577 subjects without diabetes [18]. During the 4-year follow-up, they found that TC/HDL-C ratio had the best performance in reflecting future diabetes risk. It should be noted that in the study of Professor Hadaegh and Professor Seo [18, 19], despite their follow-up, the time factor was not taken into account in the final data analysis. The results of their final analysis were based on multivariate Logistic regression analysis, which may be biased. Recently, a longitudinal study by Khaloo et al. involving 5474 non-diabetic subjects showed that Ln-TG/HDL-C ratio was a better lipid parameter to predict future diabetes risk [10]. Summing up the results of several similar studies above, among multiple lipid parameters, the TC/HDL-C ratio and TG/HDL-C ratio may be good parameters for predicting future diabetes. In our current study, we included more unconventional lipid parameters in a cohort of 15,464 participants with baseline normoglycemia. During a follow-up of up to 13 years, we further confirmed their findings: both TC/HDL-C ratio and TG/HDL-C ratio were good lipid parameters for predicting future diabetes risk. On this basis, our results added more evidence, and we found that among all lipid parameters, the RC/HDL-C ratio was the best parameter to reflect the risk of developing diabetes in the future. This finding has not been reported in previous similar studies, and so far as we know, this is the first study to evaluate the relationship between diabetes and RC/HDL-C ratio.

Several studies have been reported on the thresholds of conventional versus unconventional lipid parameters for the diagnosis/prediction of diabetes [9, 18, 49, 50]. In summary, the main differences between the results of the threshold analysis of lipid parameters in the published studies and the analysis in our current study are in the TG and the consideration of time variables. Compared with other threshold analysis studies, our current study fully considered time-dependent variables and assessed baseline lipid parameters as predictors of future diabetes risk, while the existing similar studies focused more on the present [9, 18, 49, 50]. Furthermore, in the current study, we also evaluated the predictive value of conventional and unconventional lipid parameters for predicting short -, medium-and long-term diabetes risk. Overall, the RC/HDL-C ratio had the best predictive value for predicting the short-term diabetes risk, non-HDL/HDL-C ratio and LDL/HDL-C ratio for the short-to medium-term diabetes risk, while RC, non-HDL/HDL-C ratio, and TC/HDL-C ratio for the mid-to long-term and long-term diabetes risk.

Conventional methods for measuring lipids and lipoproteins include chemical methods and enzymatic methods. Generally speaking, chemical methods are relatively time-consuming, so they are usually only used for calibration in the clinic, while the enzyme method has been used as the most commonly used analysis method in automatic biochemical analytical instruments due to its relative simplicity and accuracy [51, 52]. Although the conventional lipid measurement methods have excellent diagnostic performance, they still require expensive instruments, trained operators, and dedicated laboratories. Therefore, conventional lipid measurement methods may not be optimal for primary health care institutions and people with limited mobility [53]. Recently, biosensors for measuring lipids and lipoproteins have been developed for preventive and therapeutic monitoring of chronic diseases at the proof-of-concept stage, allowing simultaneous measurement of multiple lipid parameters in a home setting by a user with minimal training [54–56]. Compared with conventional methods, biosensors for lipids and lipoproteins are more convenient, rapid, and easy to operate. In addition, recent studies have shown that new biomaterials (such as nanomaterials) have better biocompatibility, stability, and unique physical, electronic and chemical properties [52, 57, 58]. These new materials and techniques may have great potential for early screening of people at high risk of diabetes by testing lipid parameters [59].

There are many mechanisms by which lipid parameters are associated with diabetes. From a clinical point of view, the atherogenic effect of lipid parameters and IR caused by lipid parameters may be the main factors associated with diabetes [4, 60–62]. Among them, the vasoactive hormone pathway including the renin–angiotensin–aldosterone system (RAAS) seems to play an important role in this process [63]. The RAAS is known to be an important system in the body for maintaining plasma sodium concentration, arterial blood pressure, and extracellular volume [64]. The imbalance between renin and angiotensin II can lead to a large number of chronic and acute diseases [64, 65], and plaque formation induced by angiotensin II in the early stage is one of the most important effects of RAAS on atherosclerosis [66], while under pathological conditions, RAAS also contributes directly or indirectly to the development of atherosclerosis and its various complications through its effects on other systems [67]. In addition to this, when renin and angiotensin II are imbalanced, the RAAS detrimental axis will also increase the release of inflammatory cytokines, and generate and increase oxidative stress; these pathological changes will further promote the formation of atherosclerosis, exacerbated IR, and decreased insulin secretion [66, 68]. On the other hand, it should also be noted that serum hepcidin and hepcidin/ferritin ratio also play an important role in the development of IR and diabetes [69, 70]. In the current research, we calculated the IR substitute index METS-IR, and the correlation analysis showed that all lipid parameters were closely related to METS-IR, among which TG/HDL-C ratio had the strongest correlation (Pearson r = 0.7447 for TG/HDL-C ratio). Additionally, it is worth noting that unconventional lipid parameters were also more powerful in identifying atherosclerosis and IR than conventional lipid parameters [61, 71]. Combining current research findings, we have several simple suggestions for a series of possible future work: (1) it is suggested that medical staff should strengthen their understanding of unconventional lipid parameters. (2) It is suggested that based on conventional lipid measurement, the inspection center of medical institutions can add unconventional lipid parameters as detection items to the display list by adding an algorithm to the computer. (3) It is suggested that more comparative studies on conventional and unconventional lipid parameters should be carried out to find out the value of lipid parameters in the risk assessment of other diseases. (4) It is suggested to add more unconventional lipid parameters on the basis of non-HDL-C as the main target of lipid management [72]. (5) It is suggested that unconventional lipid parameters should be considered as a potential target for developing high-sensitivity biosensors for diabetes.

### Advantages and limitations of research

While interpreting the current research results, there are several research advantages to be mentioned: (1) Compared with similar studies, the current study further expanded the sample size based on longitudinal design and included more unconventional lipid parameters. (2) We investigated the relationship of RC/HDL-C ratio with diabetes for the first time, and through multivariate Cox regression analysis found that compared with other conventional and unconventional lipid parameters, the RC/HDL-C ratio could better reflect the future risk of diabetes. (3) The current study compared the predictive value of lipid parameters for future diabetes by time-dependent ROC curve analysis for the first time.

Several research limitations need to be acknowledged: (1) the endpoint of the current study is new-onset diabetes events, while the death events during follow-up are not recorded in the current dataset, which may have a certain competitive risk to the current research results. (2) In this study, we analyzed the predictive value of several lipid parameters for the risk of developing diabetes in 3, 6, 9, and 12 years respectively, but the results of the current study may be more suitable for the short-and medium-term risk prediction of diabetes because the body’s metabolic profile gradually deteriorates with age [73], the previously predicted risk of diabetes may no longer apply. (3) The participants of the current study were ordinary people who underwent health check-ups. Generally speaking, most people who receive a health examination do not have a routine measurement of postprandial glucose. Therefore, the current study may underestimate the incidence of diabetes. (4) The types of diabetes have not been distinguished in the current study, but based on a large number of published research data [1, 8, 74], the current results are more suitable for type 2 diabetes, and the applicability in other special types of diabetes needs further study.

### Conclusion

In summary, our results demonstrated the importance of unconventional lipid parameters for predicting the risk of diabetes in the future. Compared with conventional lipid parameters, it is worthwhile to use unconventional lipid parameters to predict the future risk of diabetes. It is recommended to incorporate unconventional lipid parameters as soon as possible in clinical practice for routine assessment of diabetes risk and treatment monitoring.

## Supplementary Information

**Additional file 1: Text S1.** STROBE Statement—checklist of items that should be included in reports of observational studies.

**Additional file 2: Figure S1.** Proportional hazards assumption checking. **Figure S2. A** RCS evaluates the best threshold of HDL-C for predicting future diabetes risk. **B** RCS evaluates the best threshold of TG for predicting future diabetes risk. **C** RCS evaluates the best threshold of RC for predicting future diabetes risk. **D** RCS evaluates the best threshold of TC/HDL-C ratio for predicting future diabetes risk. **E** RCS evaluates the best threshold of TG/HDL-C ratio for predicting future diabetes risk. **F** RCS evaluates the best threshold of LDL/HDL-C ratio for predicting future diabetes risk. **G** RCS evaluates the best threshold of non-HDL/HDL-C ratio for predicting future diabetes risk. **H** RCS evaluates the best threshold of RC/HDL-C ratio for predicting future diabetes risk.

**Additional file 3: Table S1.** Pearson correlation analysis of baseline conventional and unconventional lipid parameters and METS-IR. **Table S2.** Collinearity diagnostics steps.

## Data Availability

The data used in this study have been uploaded to the “Dryad” database by Professor Okamura et al.

## Abbreviations

TC: Total cholesterol
TG: Triglyceride
HDL-C: High-density lipoprotein cholesterol
LDL-C: Low-density lipoprotein cholesterol
RC: Remnant cholesterol
HR: Hazard ratio
CI: Confidence interval
ROC: Receiver operator characteristics
BMI: Body mass index
AST: Aspartate aminotransferase
GGT: Gamma-glutamyl transferase
ALT: Alanine aminotransferase
FPG: Fasting plasma glucose
HbA1c: Hemoglobin A1c
METS-IR: Metabolic score for insulin resistance
VIF: Variance inflation factor
RCS: Restricted cubic splines
KM: Kaplan–Meier

## References

[1] Saeedi P, Petersohn I, Salpea P, Malanda B, Karuranga S, Unwin N (2019). Global and regional diabetes prevalence estimates for 2019 and projections for 2030 and 2045: results from the International Diabetes Federation Diabetes Atlas, 9th edition. Diabetes Res Clin Pract.

[2] Mortality and Causes of Death Collaborators (2016). Global, regional, and national life expectancy, all-cause mortality, and cause-specific mortality for 249 causes of death, 1980–2015: a systematic analysis for the Global Burden of Disease Study 2015. Lancet.

[3] GBD 2015 DALYs and HALE Collaborators (2016). Global, regional, and national disability-adjusted life-years (DALYs) for 315 diseases and injuries and healthy life expectancy (HALE), 1990–2015: a systematic analysis for the Global Burden of Disease Study 2015. Lancet.

[4] Krauss RM (2004). Lipids and lipoproteins in patients with type 2 diabetes. Diabetes Care.

[5] Sascău R, Clement A, Radu R, Prisacariu C, Stătescu C (2021). Triglyceride-rich lipoproteins and their remnants as silent promoters of atherosclerotic cardiovascular disease and other metabolic disorders: a review. Nutrients.

[6] Borén J, Taskinen MR, Björnson E, Packard CJ (2022). Metabolism of triglyceride-rich lipoproteins in health and dyslipidaemia. Nat Rev Cardiol.

[7] Krauss RM (1998). Triglycerides and atherogenic lipoproteins: rationale for lipid management. Am J Med.

[8] Yang W, Lu J, Weng J, Jia W, Ji L, Xiao J (2010). Prevalence of diabetes among men and women in China. N Engl J Med.

[9] Xie G, Zhong Y, Yang S, Zou Y (2021). Remnant cholesterol is an independent predictor of new-onset diabetes: a single-center cohort study. Diabetes Metab Syndr Obes.

[10] Khaloo P, Hasheminia M, Tohidi M, Abdi H, Mansournia MA, Azizi F (2018). Impact of 3-year changes in lipid parameters and their ratios on incident type 2 diabetes: Tehran lipid and glucose study. Nutr Metab.

[11] Zhang N, Hu X, Zhang Q, Bai P, Cai M, Zeng TS (2018). Non-high-density lipoprotein cholesterol: high-density lipoprotein cholesterol ratio is an independent risk factor for diabetes mellitus: results from a population-based cohort study. J Diabetes.

[12] Hong M, Ling Y, Lu Z, Liu Y, Gu P, Shao J (2019). Contribution and interaction of the low-density lipoprotein cholesterol to high-density lipoprotein cholesterol ratio and triglyceride to diabetes in hypertensive patients: a cross-sectional study. J Diabetes Investig.

[13] van Wijk DF, Stroes ES, Kastelein JJ (2009). Lipid measures and cardiovascular disease prediction. Dis Markers.

[14] Manickam P, Rathod A, Panaich S, Hari P, Veeranna V, Badheka A (2011). Comparative prognostic utility of conventional and novel lipid parameters for cardiovascular disease risk prediction: do novel lipid parameters offer an advantage?. J Clin Lipidol.

[15] Zhu L, Lu Z, Zhu L, Ouyang X, Yang Y, He W (2015). Lipoprotein ratios are better than conventional lipid parameters in predicting coronary heart disease in Chinese Han people. Kardiol Pol.

[16] Tancredi M, Rosengren A, Svensson AM, Kosiborod M, Pivodic A, Gudbjörnsdottir S (2015). Excess mortality among persons with type 2 diabetes. N Engl J Med.

[17] Chen Z, Hu H, Chen M, Luo X, Yao W, Liang Q (2020). Association of triglyceride to high-density lipoprotein cholesterol ratio and incident of diabetes mellitus: a secondary retrospective analysis based on a Chinese cohort study. Lipids Health Dis.

[18] Seo MH, Bae JC, Park SE, Rhee EJ, Park CY, Oh KW (2011). Association of lipid and lipoprotein profiles with future development of type 2 diabetes in nondiabetic Korean subjects: a 4-year retrospective, longitudinal study. J Clin Endocrinol Metab.

[19] Hadaegh F, Hatami M, Tohidi M, Sarbakhsh P, Saadat N, Azizi F (2010). Lipid ratios and appropriate cut off values for prediction of diabetes: a cohort of Iranian men and women. Lipids Health Dis.

[20] Okamura T (2019). Dryad Dataset.

[21] Okamura T, Hashimoto Y, Hamaguchi M, Obora A, Kojima T, Fukui M (2019). Ectopic fat obesity presents the greatest risk for incident type 2 diabetes: a population-based longitudinal study. Int J Obes.

[22] Hashimoto Y, Hamaguchi M, Kojima T, Ohshima Y, Ohbora A, Kato T (2015). Modest alcohol consumption reduces the incidence of fatty liver in men: a population-based large-scale cohort study. J Gastroenterol Hepatol.

[23] Chen Y, Zhang X, Pan B, Jin X, Yao H, Chen B (2010). A modified formula for calculating low-density lipoprotein cholesterol values. Lipids Health Dis.

[24] Nordestgaard BG, Varbo A (2014). Triglycerides and cardiovascular disease. Lancet.

[25] Zou Y, Hu C, Kuang M, Chai Y (2022). Remnant cholesterol/high-density lipoprotein cholesterol ratio is a new powerful tool for identifying non-alcoholic fatty liver disease. BMC Gastroenterol.

[26] American Diabetes Association (2011). Standards of medical care in diabetes—2011. Diabetes Care.

[27] Hamaguchi M, Kojima T, Itoh Y, Harano Y, Fujii K, Nakajima T (2007). The severity of ultrasonographic findings in nonalcoholic fatty liver disease reflects the metabolic syndrome and visceral fat accumulation. Am J Gastroenterol.

[28] Bello-Chavolla OY, Almeda-Valdes P, Gomez-Velasco D, Viveros-Ruiz T, Cruz-Bautista I, Romo-Romo A (2018). METS-IR, a novel score to evaluate insulin sensitivity, is predictive of visceral adiposity and incident type 2 diabetes. Eur J Endocrinol.

[29] Sato T, Matsuyama Y (2003). Marginal structural models as a tool for standardization. Epidemiology.

[30] Muanda FT, Weir MA, Bathini L, Blake PG, Chauvin K, Dixon SN (2019). Association of baclofen with encephalopathy in patients with chronic kidney disease. JAMA.

[31] Box GEP, Cox DR (1964). An analysis of transformations. J R Stat Soc Ser B.

[32] Kim JH (2019). Multicollinearity and misleading statistical results. Korean J Anesthesiol.

[33] Fitchett EJA, Seale AC, Vergnano S, Sharland M, Heath PT, Saha SK (2016). Strengthening the reporting of observational studies in epidemiology for newborn infection (STROBE-NI): an extension of the STROBE statement for neonatal infection research. Lancet Infect Dis.

[34] Durrleman S, Simon R (1989). Flexible regression models with cubic splines. Stat Med.

[35] Stidsen JV, Henriksen JE, Olsen MH, Thomsen RW, Nielsen JS, Rungby J (2018). Pathophysiology-based phenotyping in type 2 diabetes: a clinical classification tool. Diabetes Metab Res Rev.

[36] Wagner R, Heni M, Tabák AG, Machann J, Schick F, Randrianarisoa E (2021). Pathophysiology-based subphenotyping of individuals at elevated risk for type 2 diabetes. Nat Med.

[37] Kendall DM (2005). The dyslipidemia of diabetes mellitus: giving triglycerides and high-density lipoprotein cholesterol a higher priority?. Endocrinol Metab Clin North Am.

[38] Alexopoulos AS, Qamar A, Hutchins K, Crowley MJ, Batch BC, Guyton JR (2019). Triglycerides: emerging targets in diabetes care? Review of moderate hypertriglyceridemia in diabetes. Curr Diab Rep.

[39] Bitzur R, Cohen H, Kamari Y, Shaish A, Harats D (2009). Triglycerides and HDL cholesterol: stars or second leads in diabetes?. Diabetes Care.

[40] Pan W, Sun W, Yang S, Zhuang H, Jiang H, Ju H (2020). LDL-C plays a causal role on T2DM: a Mendelian randomization analysis. Aging.

[41] Cui J, Ma P, Sun JP, Baloch Z, Yin F, Xin HL (2019). The ability of baseline triglycerides and total cholesterol concentrations to predict incidence of type 2 diabetes mellitus in chinese men and women: a longitudinal study in Qingdao, China. Biomed Environ Sci.

[42] Shi Q, Liu S, Krousel-Wood M, Shao H, Fonseca V, Shi L (2018). Long-term outcomes associated with triple-goal achievement in patients with type 2 diabetes mellitus (T2DM). Diabetes Res Clin Pract.

[43] Taskinen MR, Barter PJ, Ehnholm C, Sullivan DR, Mann K, Simes J (2010). Ability of traditional lipid ratios and apolipoprotein ratios to predict cardiovascular risk in people with type 2 diabetes. Diabetologia.

[44] Liu X, Yan L, Xue F (2019). The associations of lipids and lipid ratios with stroke: a prospective cohort study. J Clin Hypertens.

[45] Sheng G, Lu S, Xie Q, Peng N, Kuang M, Zou Y (2021). The usefulness of obesity and lipid-related indices to predict the presence of non-alcoholic fatty liver disease. Lipids Health Dis.

[46] Yan X, Gao Y, Zhao Q, Qiu X, Tian M, Dai J (2021). Correlation of lipid ratios with the severity of pulmonary alveolar proteinosis: a cross-sectional study. Front Nutr.

[47] Zhang L, Yuan Z, Chen W, Chen S, Liu X, Liang Y (2014). Serum lipid profiles, lipid ratios and chronic kidney disease in a Chinese population. Int J Environ Res Public Health.

[48] Yu Y, Lan T, Wang D, Fang W, Tao Y, Li M (2021). The association of lipid ratios with hyperuricemia in a rural Chinese hypertensive population. Lipids Health Dis.

[49] Zhou Y, Yang G, Qu C, Chen J, Qian Y, Yuan L (2022). Predictive performance of lipid parameters in identifying undiagnosed diabetes and prediabetes: a cross-sectional study in eastern China. BMC Endocr Disord.

[50] Song Q, Liu X, Wang A, Wang Y, Zhou Y, Zhou W (2016). Associations between non-traditional lipid measures and risk for type 2 diabetes mellitus in a Chinese community population: a cross-sectional study. Lipids Health Dis.

[51] Nakamura M, Iso H, Kitamura A, Imano H, Kiyama M, Yokoyama S (2015). Total cholesterol performance of Abell-Levy-Brodie-Kendall reference measurement procedure: Certification of Japanese in-vitro diagnostic assay manufacturers through CDC’s Cholesterol Reference Method Laboratory Network. Clin Chim Acta.

[52] Lu S, Yu T, Wang Y, Liang L, Chen Y, Xu F (2017). Nanomaterial-based biosensors for measurement of lipids and lipoproteins towards point-of-care of cardiovascular disease. Analyst.

[53] Qureshi A, Gurbuz Y, Niazi JH (2012). Biosensors for cardiac biomarkers detection: a review. Sens Actuators.

[54] Sekretaryova AN, Eriksson M, Turner AP (2016). Bioelectrocatalytic systems for health applications. Biotechnol Adv.

[55] Saxena U, Das AB (2016). Nanomaterials towards fabrication of cholesterol biosensors: key roles and design approaches. Biosens Bioelectron.

[56] Ferreira CE, França CN, Correr CJ, Zucker ML, Andriolo A, Scartezini M (2015). Clinical correlation between a point-of-care testing system and laboratory automation for lipid profile. Clin Chim Acta.

[57] Suhito IR, Koo KM, Kim TH (2020). Recent advances in electrochemical sensors for the detection of biomolecules and whole cells. Biomedicines.

[58] Howes PD, Chandrawati R, Stevens MM (2014). Colloidal nanoparticles as advanced biological sensors. Science.

[59] Salek-Maghsoudi A, Vakhshiteh F, Torabi R, Hassani S, Ganjali MR, Norouzi P (2018). Recent advances in biosensor technology in assessment of early diabetes biomarkers. Biosens Bioelectron.

[60] Kane JP, Pullinger CR, Goldfine ID, Malloy MJ (2021). Dyslipidemia and diabetes mellitus: role of lipoprotein species and interrelated pathways of lipid metabolism in diabetes mellitus. Curr Opin Pharmacol.

[61] Zhang L, Chen S, Deng A, Liu X, Liang Y, Shao X (2015). Association between lipid ratios and insulin resistance in a Chinese population. PLoS ONE.

[62] Poznyak A, Grechko AV, Poggio P, Myasoedova VA, Alfieri V, Orekhov AN (2020). The diabetes mellitus-atherosclerosis connection: the role of lipid and glucose metabolism and chronic inflammation. Int J Mol Sci.

[63] Jandeleit-Dahm K, Cooper ME (2006). Hypertension and diabetes: role of the renin-angiotensin system. Endocrinol Metab Clin North Am.

[64] Patel S, Rauf A, Khan H, Abu-Izneid T (2017). Renin-angiotensin-aldosterone (RAAS): the ubiquitous system for homeostasis and pathologies. Biomed Pharmacother.

[65] Ahmadian E, Pennefather PS, Eftekhari A, Heidari R, Eghbal MA (2016). Role of renin-angiotensin system in liver diseases: an outline on the potential therapeutic points of intervention. Expert Rev Gastroenterol Hepatol.

[66] Poznyak AV, Bharadwaj D, Prasad G, Grechko AV, Sazonova MA, Orekhov AN (2021). Renin-angiotensin system in pathogenesis of atherosclerosis and treatment of CVD. Int J Mol Sci.

[67] Durante A, Peretto G, Laricchia A, Ancona F, Spartera M, Mangieri A (2012). Role of the renin-angiotensin-aldosterone system in the pathogenesis of atherosclerosis. Curr Pharm Des.

[68] Favre GA, Esnault VL, Van Obberghen E (2015). Modulation of glucose metabolism by the renin-angiotensin-aldosterone system. Am J Physiol Endocrinol Metab.

[69] Aregbesola A, Voutilainen S, Virtanen JK, Aregbesola A, Tuomainen TP (2015). Serum hepcidin concentrations and type 2 diabetes. World J Diabetes.

[70] Karamzad N, Eftekhari A, Ashrafi-Asgarabad A, Sullman MJM, Sahebkar A, Safiri S (2021). Serum Hepcidin, the hepcidin/ferritin ratio and the risk of type 2 diabetes: a systematic review and meta-analysis. Curr Med Chem.

[71] Yang WS, Li R, Shen YQ, Wang XC, Liu QJ, Wang HY (2020). Importance of lipid ratios for predicting intracranial atherosclerotic stenosis. Lipids Health Dis.

[72] Osentino F, Grant PJ, Aboyans V, Bailey CJ, Ceriello A, Delgado V (2020). 2019 ESC Guidelines on diabetes, pre-diabetes, and cardiovascular diseases developed in collaboration with the EASD. Eur Heart J.

[73] Varghese M, Song J, Singer K (2021). Age and sex: impact on adipose tissue metabolism and inflammation. Mech Ageing Dev.

[74] Neville SE, Boye KS, Montgomery WS, Iwamoto K, Okamura M, Hayes RP (2009). Diabetes in Japan: a review of disease burden and approaches to treatment. Diabetes Metab Res Rev.

