# Supplementary material for: Evaluation of the value of conventional and unconventional lipid parameters for predicting the risk of diabetes in a non-diabetic population
Source: J Transl Med. 2022 Jun 11;20:266. doi: 10.1186/s12967-022-03470-z (PMC9188037; doi:10.1186/s12967-022-03470-z)
Supplement: Supplementary file 2 — Additional file 2: Figure S1. Proportional hazards assumption checking. Figure S2. A RCS evaluates the best threshold of HDL-C for predicting future diabetes risk. B RCS evaluates the best threshold of TG for predicting future diabetes risk. C RCS evaluates the best threshold of RC for predicting future diabetes risk. D RCS evaluates the best threshold of TC/HDL-C ratio for predicting future diabetes risk. E RCS evaluates the best threshold of TG/HDL-C ratio for predicting future diabetes risk. F RCS evaluates the best threshold of LDL/HDL-C ratio for predicting future diabetes risk. G RCS evaluates the best threshold of non-HDL/HDL-C ratio for predicting future diabetes risk. H RCS evaluates the best threshold of RC/HDL-C ratio for predicting future diabetes risk. [file 12967_2022_3470_MOESM2_ESM.pdf]

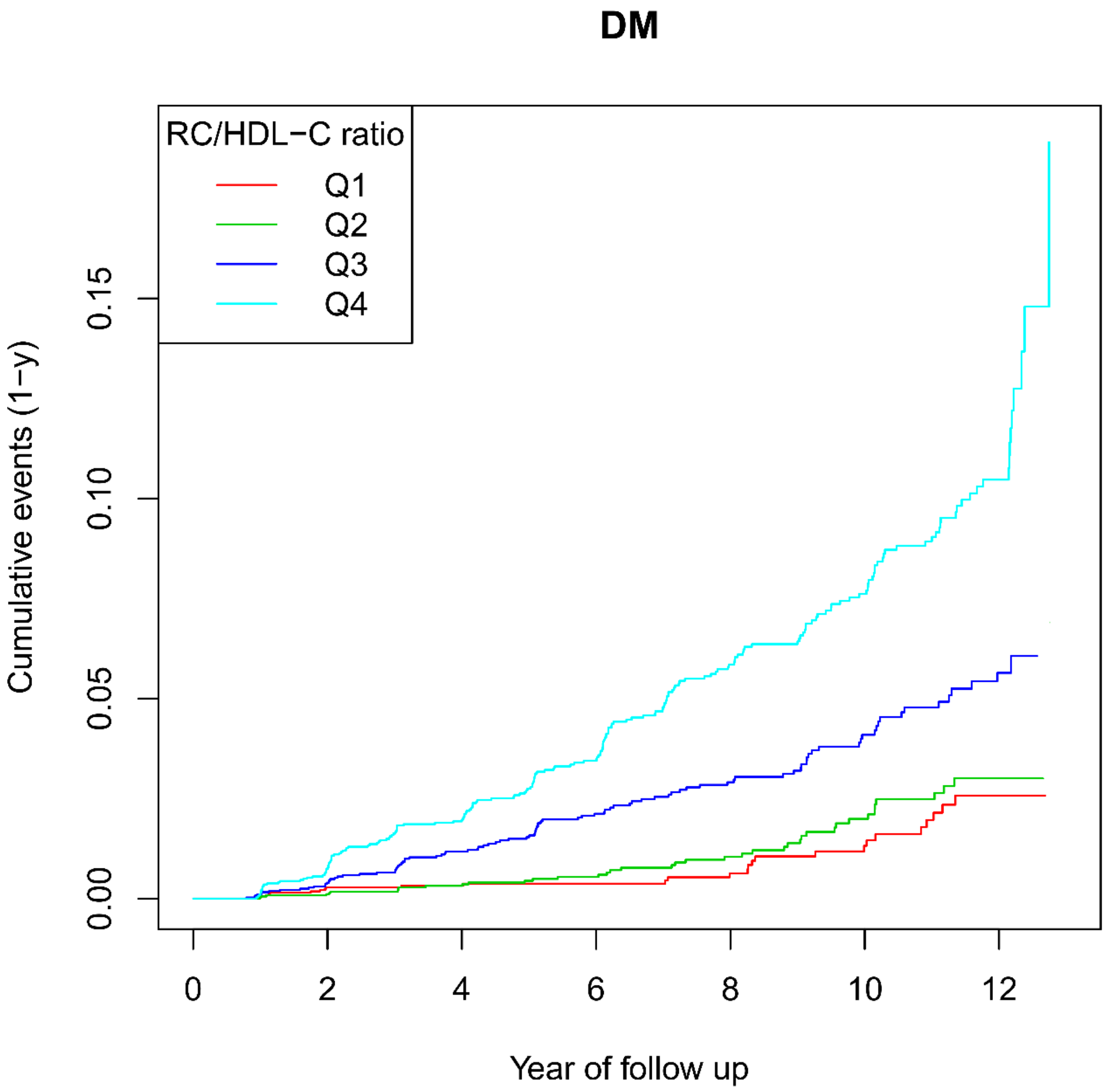

Figure S1: Proportional hazards assumption checking.

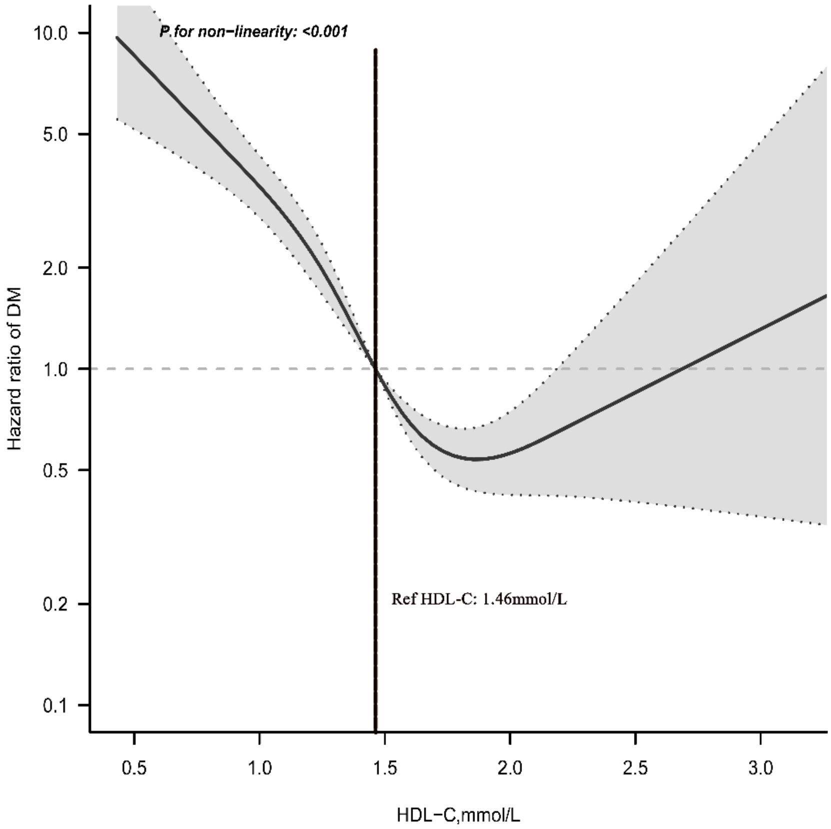

Figure S2(A): RCS evaluates the best threshold of HDL-C for predicting future diabetes risk.

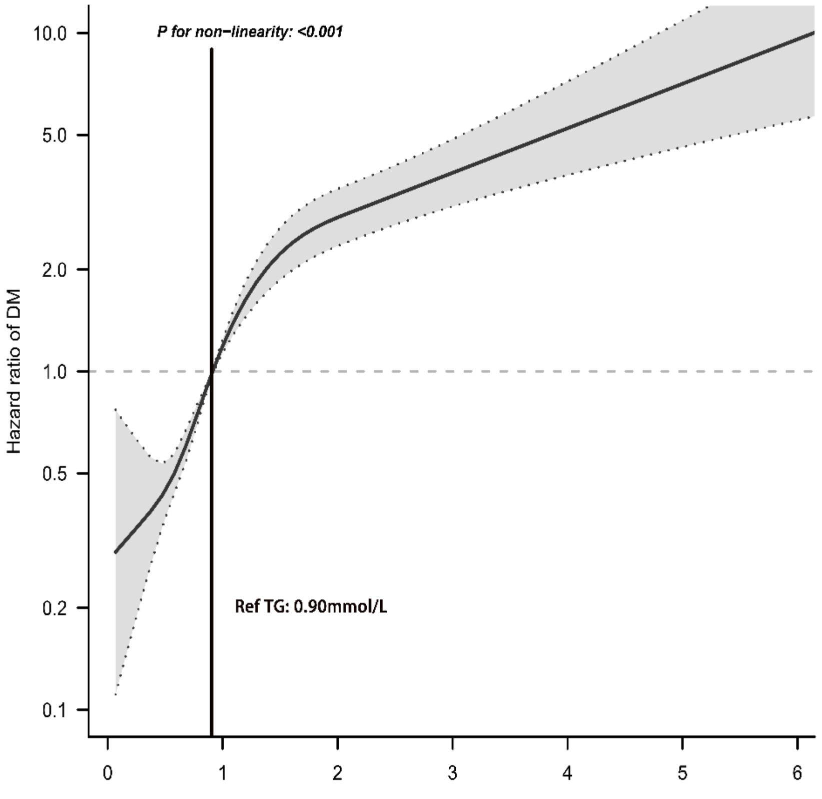

Figure S2(B): RCS evaluates the best threshold of TG for predicting future diabetes risk.

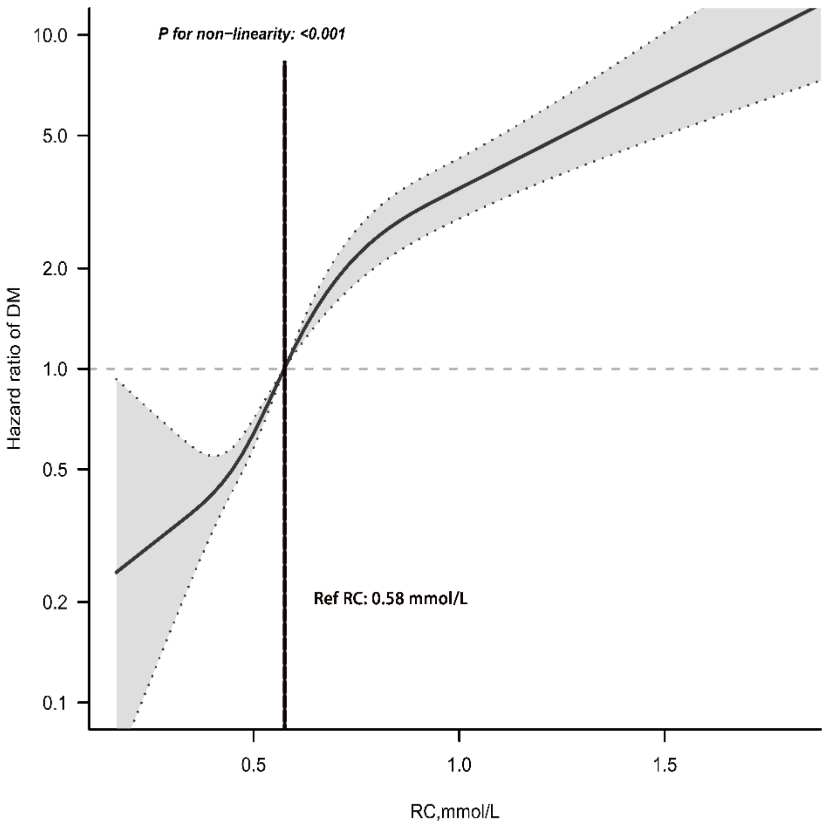

Figure S2(C): RCS evaluates the best threshold of RC for predicting future diabetes risk.

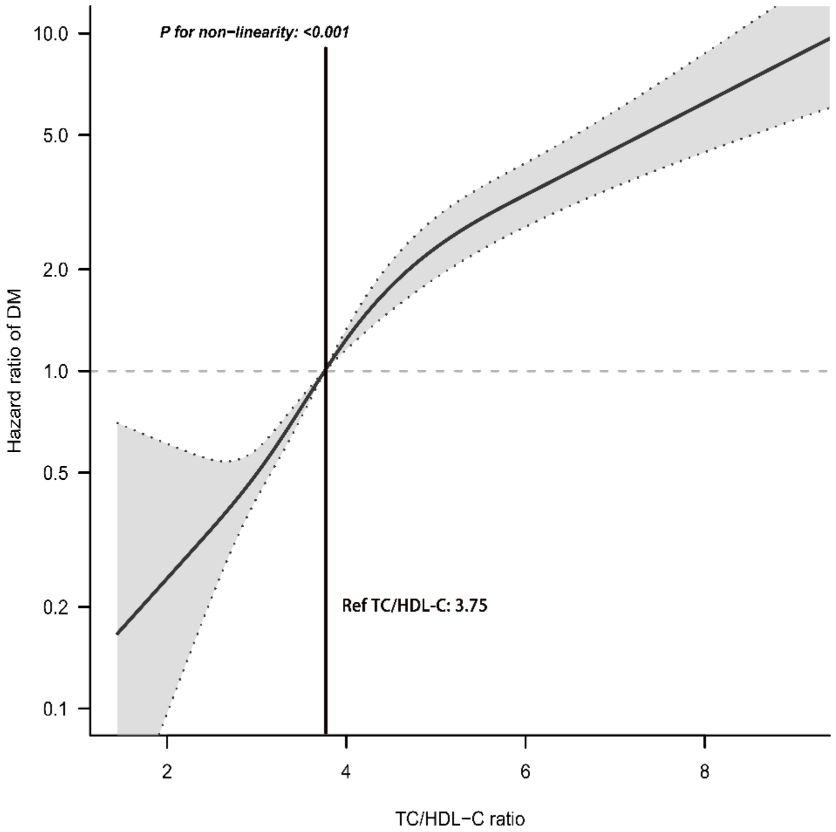

Figure S2(D): RCS evaluates the best threshold of TC/HDL-C ratio for predicting future diabetes risk.

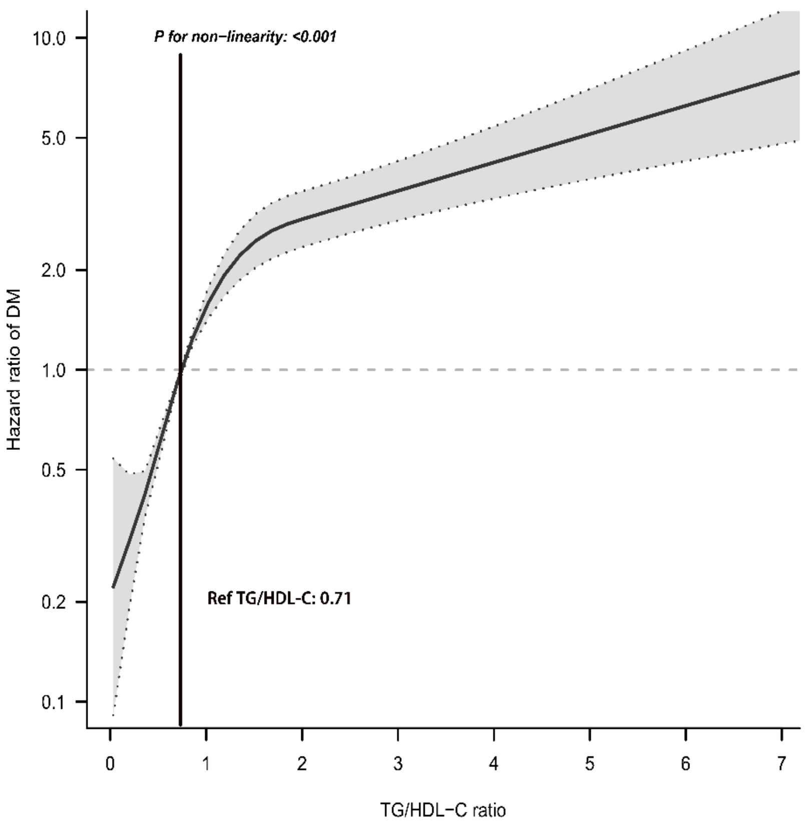

Figure S2(E): RCS evaluates the best threshold of TG/HDL-C ratio for predicting future diabetes risk.

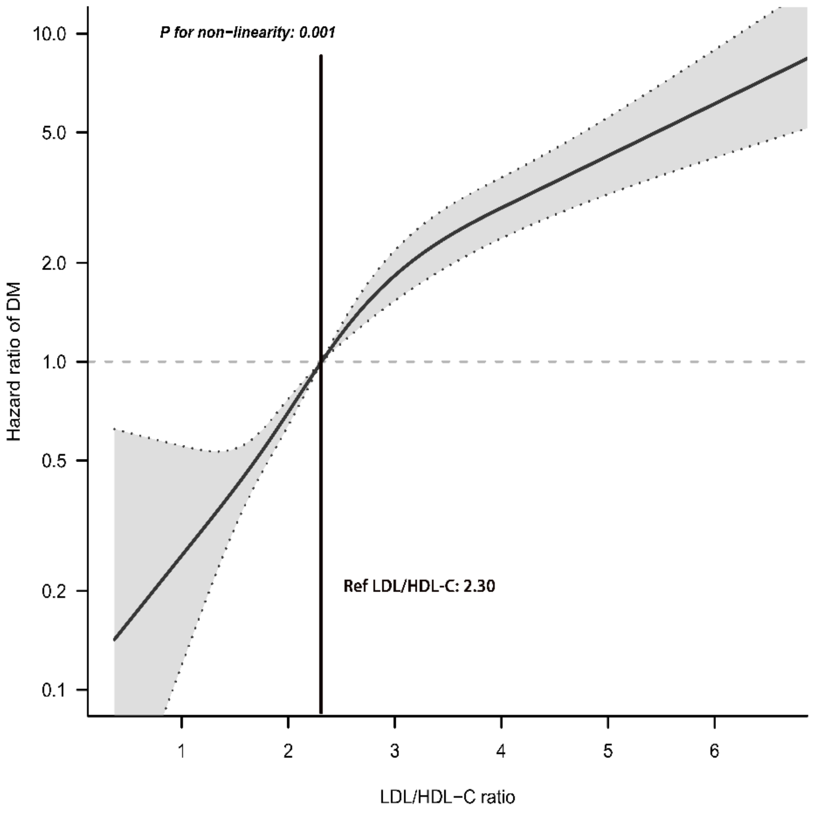

Figure S2(F): RCS evaluates the best threshold of LDL/HDL-C ratio for predicting future diabetes risk.

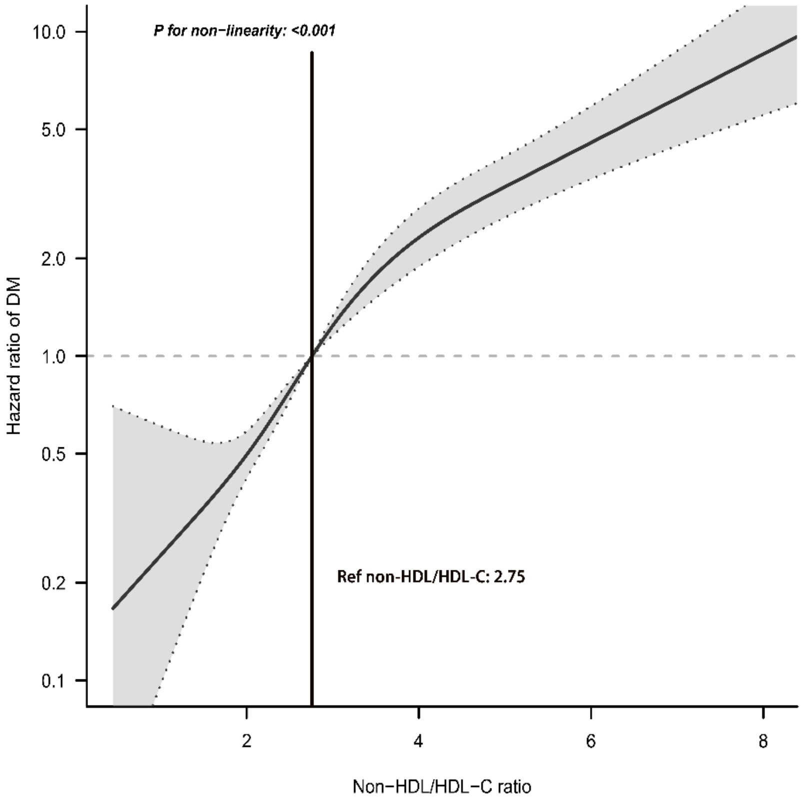

Figure S2(G): RCS evaluates the best threshold of non-HDL/HDL-C ratio for predicting future diabetes risk.

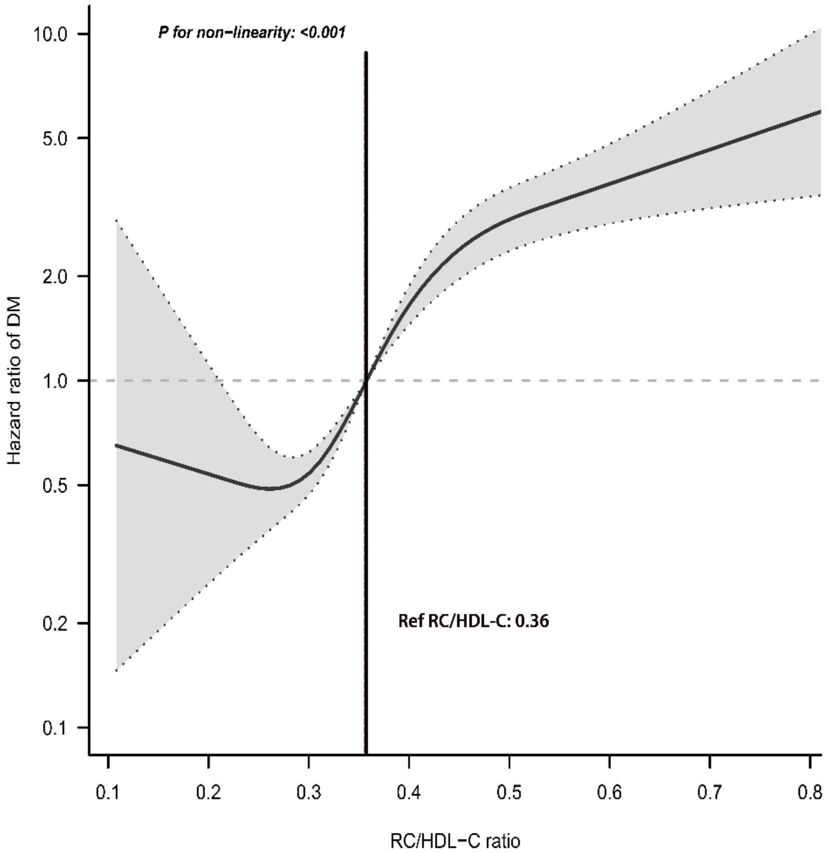

Figure S2(H): RCS evaluates the best threshold of RC/HDL-C ratio for predicting future diabetes risk.
