# Supplementary material for: Evaluation of the value of conventional and unconventional lipid parameters for predicting the risk of diabetes in a non-diabetic population
Source: J Transl Med. 2022 Jun 11;20:266. doi: 10.1186/s12967-022-03470-z (PMC9188037; doi:10.1186/s12967-022-03470-z)
Supplement: Supplementary file 3 — Additional file 3: Table S1. Pearson correlation analysis of baseline conventional and unconventional lipid parameters and METS-IR. Table S2. Collinearity diagnostics steps. [file 12967_2022_3470_MOESM3_ESM.pdf]

Table S1: Pearson correlation Analysis of baseline conventional and unconventional lipid parameters and METS-IR.

	Correlation	95%CI low	95%CI upp	*P*-value
HDL-C	-0.7139	-0.7216	-0.7061	<0.0001
TG	0.6460	0.6367	0.6551	<0.0001
TC	0.1680	0.1526	0.1833	<0.0001
LDL-C	0.3823	0.3687	0.3956	<0.0001
RC	0.6383	0.6289	0.6476	<0.0001
Non-HDL/C ratio	0.4653	0.4529	0.4776	<0.0001
TC/HDL-C ratio	0.7324	0.7250	0.7396	<0.0001
TG/HDL-C ratio	0.7447	0.7376	0.7517	<0.0001
Non-HDL/HDL-C ratio	0.7318	0.7244	0.7390	<0.0001
LDL/HDL-C ratio	0.7049	0.6969	0.7128	<0.0001
RC/HDL-C ratio	0.7139	0.7061	0.7216	<0.0001

Abbreviations as in Table 1.

Table S2: Collinearity diagnostics steps.

	VIF											
	Step 1	Step 2	Step 3	Step 4	Step 5	Step 6	Step 7	Step 8	Step 9	Step 10	Step 11	Step 12
Sex	3.3	3.3	3.3	3.3	3.3	3.3	3.2	3.2	3.2	3.2	3.2	3.2
Age	1.4	1.4	1.4	1.4	1.4	1.4	1.4	1.4	1.4	1.4	1.3	1.3
Fatty liver	1.6	1.6	1.6	1.6	1.6	1.6	1.6	1.6	1.6	1.6	1.6	1.6
Height	52.9	52.9	52.9	52.9	52.9	52.9	52.9	2.8	2.8	2.8	2.4	2.4
Weight	170.9	170.9	170.9	170.9	170.9	170.9	170.9	NA	NA	NA	NA	NA
BMI	96.9	96.9	96.9	96.9	96.9	96.9	96.9	5.1	5	5	1.8	1.8
WC	6	6	6	6	6	6	6	6	6	6	NA	NA
ALT	4.2	4.2	4.2	4.2	4.2	4.2	4.2	4.2	4.2	4.2	4.2	4.1
AST	3.3	3.3	3.3	3.3	3.3	3.3	3.3	3.3	3.3	3.3	3.3	3.3
GGT	1.5	1.5	1.5	1.5	1.5	1.5	1.5	1.5	1.5	1.5	1.5	1.5
HDL-C	Inf	NA	NA	NA	NA	NA	NA	NA	NA	NA	NA	NA
TC	Inf	47.8	47.8	47.8	47.8	47.8	39	38.9	17	2.7	2.7	2.7
TG	Inf	Inf	NA	NA	NA	NA	NA	NA	NA	NA	NA	NA
LDL-C	Inf	Inf	Inf	NA	NA	NA	NA	NA	NA	NA	NA	NA
Non-HDL-C	Inf	Inf	Inf	86.6	86.6	86.6	50.7	50.4	NA	NA	NA	NA
RC	Inf	Inf	Inf	40.5	40.5	40.5	4.9	4.9	4.6	3.8	3.8	3.8
TC/HDL-C ratio	Inf	Inf	Inf	Inf	NA	NA	NA	NA	NA	NA	NA	NA
TG/HDL-C ratio	Inf	Inf	Inf	Inf	Inf	NA	NA	NA	NA	NA	NA	NA
LDL/HDL-C ratio	Inf	Inf	Inf	Inf	Inf	828.8	40.7	40.7	32.4	NA	NA	NA
Non-HDL/HDL-C ratio	Inf	Inf	Inf	Inf	Inf	979.4	NA	NA	NA	NA	NA	NA
RC/HDL-C ratio	38	38	38	38	38	38	31.5	31.5	31.5	2.5	2.5	2.5
FPG	1.5	1.5	1.5	1.5	1.5	1.5	1.5	1.5	1.5	1.5	1.5	1.5
HbA1c	1.3	1.3	1.3	1.3	1.3	1.3	1.3	1.3	1.3	1.3	1.3	1.2
SBP	5.6	5.6	5.6	5.6	5.6	5.6	5.6	5.6	5.6	5.6	5.6	1.4
DBP	5.7	5.7	5.7	5.7	5.7	5.7	5.7	5.7	5.7	5.7	5.7	NA
Exercise habits	1	1	1	1	1	1	1	1	1	1	1	1
Drinking status	1.3	1.3	1.3	1.3	1.3	1.3	1.3	1.3	1.3	1.3	1.3	1.3
Smoking status	1.4	1.4	1.4	1.4	1.4	1.4	1.4	1.4	1.4	1.4	1.4	1.4

Abbreviations: Inf: infinity; VIF: Variance inflation factor; Other abbreviations as in Table 1.

Note: VIF = $1/(1-R^2)$.
